# Tourette syndrome research highlights from 2016

**DOI:** 10.12688/f1000research.12330.2

**Published:** 2017-11-29

**Authors:** Kevin J. Black

**Affiliations:** 1 Departments of Psychiatry, Neurology, Radiology, and Neuroscience, Washington University School of Medicine, St. Louis, MO, 63110, USA

**Keywords:** Tourette syndrome, tic disorders, review, animal models, genetics, pathophysiology, therapy, premonitory

## Abstract

This article presents highlights chosen from research that appeared during 2016 on Tourette syndrome and other tic disorders. Selected articles felt to represent meaningful advances in the field are briefly summarized.

## Introduction

This is the third yearly article in the Tourette syndrome Research Highlights series, intended to share and comment on scientific and clinical advances on Gilles de la Tourette syndrome (TS) and other tic disorders. The highlights from 2017 article is being drafted on the Authorea online authoring platform, and readers are encouraged to add references or give feedback on our selections using the comment feature on that page. After the calendar year ends, the article is submitted as the annual update for the Tics collection on *F1000Research*.

## Methods

A PubMed search was conducted using the search strategy “(“Tic Disorders” [MeSH] OR Tourette NOT Tourette[AU]) AND 2016[PDAT] NOT 1950:2015[PDAT]”. On 06 Jan 2017, this search returned 186 citations, none of which had first appeared in 2017. The author also identified articles or news from other sources, such as colleagues, email lists, and professional meetings. The studies cited below were selected subjectively, but guided by a personal judgment of their potential importance to the field.

## Results

### Phenomenology and natural history

***Epidemiology.*** A large, population-based health survey was explored to determine the rate of medically diagnosed TS in Canada ^1^. One in 1,000 respondents had been diagnosed with TS, with the rate higher in youth (0.60%, *vs.* 0.09% in adults), and in males (risk ratio 5.31). Importantly, those diagnosed with TS had lower total education, income and employment. The results must be interpreted in light of the fact that many people with TS remain undiagnosed, or do not receive medical care. Yang *et al.* review tic prevalence studies in China, concluding that tics occur in about 6% of children, with persistent tics in about 1.5% ^2^. The estimates for persistent tic disorders are in line with other research, but transient tic disorder is likely substantially more common ^3^.

***Environmental effects on tic severity.*** Environmental effects on tics are typical in TS; they are included in the Diagnostic Confidence Index ^4^ and have been demonstrated under careful laboratory conditions ^5, 6^. However, typical patient and clinician understanding of TS depends largely on self-report and parental symptom report. Two reports from 2016 highlight some surprising results from prospective observation. First, in one experimental design, tics became less frequent during a short-term psychosocial stressor ^7^. This finding is counterintuitive given the daily experience of patients and observations of clinicians, and supports further research in this area.

Barnea and colleagues recorded video from 41 children age 6–18 with TS in each of five common, daily-life situations ^8^. Their findings were illuminating. First, tic frequency correlated only moderately with reports of children or parents. Self-reported premonitory urges were stronger when patients were more aware of their observed tics. Tic frequency was much higher when children were watching TV and much lower when alone, compared to doing homework, receiving attention when ticcing, or talking to a stranger. The results in the previous sentence differ from typical reports to clinicians, and suggest that developing methods for tic monitoring outside the office may be important to improve reliability and ecological validity in TS research.

***Tic suppression.*** Previous studies have attempted to address TS as a generalized failure of action inhibition, but that conceptualization may cast the net too wide ^9– 11^. A 2013 publication from Wylie *et al.* ^12^ had suggested some deficit in response inhibition (action stopping) in TS. In 2016, the group reported a new study in young adults with TS, now better controlled for comorbidity ^13^. Although TS and control groups showed similar speed of cued movement and vocalization, the tic group was slower at stopping these responses.

***Sensory phenomena.*** Many patients with TS describe sensory symptoms preceding or independent of their tics. Thresholds for externally applied sensory stimuli were similar in adults with “pure” TS and controls ^14^. These results, like those of a previous study ^15^, demonstrate that the sensory symptoms of TS are central in origin, suggesting abnormalities in interoceptive awareness or central sensorimotor processing. One group recently took a new approach to studying sensation in TS based on a Theory of Event Coding ^16^. Their results suggested that details or features of percepts are less integrated in TS; this finding applied to the group as a whole rather than relating to any obvious symptom or demographic characteristic. The authors speculate as to the possible underlying neurobiology.

***Premonitory urges.*** Premonitory urges are usually reported later in life than tics are first observed. However, a large case series ( *N*>1000) from one clinic suggests that premonitory urges “emerge much earlier than previously thought”: by age 8–10, >60% of children reported premonitory urges, and >75% could suppress tics ^17^. Urges also “were found to be highly associated with ‘not just right experiences’.” The early onset of tic suppression is consistent with a report from the author’s laboratory on tic suppression in the first few months of tic disorder onset ^18^.

Brandt *et al.* ^19^ performed a careful experiment to investigate the timing of urges in relation to tics, compulsions and—as a comparison to a naturally arising urge—blinks when attempting to keep the eyes open. Another group examined tics and urge to tic at 10- to 15-second intervals in 12 patients with moderate to severe TS; different patients had quite different relationships between urge and tic timing when examined at this temporal scale ^20^. These observations show that careful phenomenological studies still have much to teach us about tic disorders.

***Other.*** The frequency of anxiety and impulsivity in TS has suggested the possibility of a deficit in emotional self-regulation in some patients. A recent study examined specific emotional regulation approaches taken by adults with TS ^21^. The TS and control groups did not differ on anxiety or depression symptom scores, but the TS group used suppression as a strategy more often than the control group.

Self-injurious behavior (SIB) is an important clinical problem in the minority of TS patients who experience it ^22^. Sambrani *et al.* ^17^ felt that their data supported lumping SIB with coprophenomena, and argue for it to “be conceptualized as a complex tic rather than a compulsion.” Others have found similar results ^23^, though other results are contradictory ^24^, one large study found SIB to fit better with ADHD symptoms ^25^, and another linked it to both OCD and ADHD ^26^.

Self-reported depressive symptoms were as common and severe in TS as in tic-free patients with major depression, though people with TS endorsed irritability more frequently ^27^. Suicidal ideation was present in at least 8% of 75 youth ages 6–18 with a tic disorder in a clinical setting, and was independent of tic severity ^28^.

### Etiology

***Genetics.*** An important, collaborative study showed that rare copy number variants (CNVs) increase the risk for TS ^29, 30^. This was true both generally for large CNVs, exceeding 1 million base pairs, and for CNVs previously linked to pathology. Specifically, *NRXN1* and *CNTN6* each conferred a substantially increased risk of TS: 20-fold and 10-fold, respectively. These associations give important impetus to research focusing on the cellular effects of these genes and perhaps related treatments. This research also confirms that the clear genetic risk for TS is spread across numerous genes, because only 1% of patients with TS carry risk alleles in these two genes.

A population-based adult twin study of tics, ADHD and OCD found that the co-occurrence of tics and OCD can be partly explained by shared etiological influences ^31^. The same is true to some extent for tics and ADHD. Another report concluded that social disinhibition in TS is heritable ^32^. More research examining sub-dimensions of these phenotypes may clarify the situation.

Another potentially useful approach to TS genetic research was illustrated by Alexander and colleagues ^33^, who sequenced candidate genes suggested by previous research in order to identify potential functional variants linked to TS.

***Environmental risk factors and epidemiology.*** Identifying specific environmental risk factors for TS is important in its own right, and also because controlling for known environmental risks can improve the power of genetic studies. Previous studies have linked TS with lower birthweight, and more recently with maternal smoking during gestation ^34– 36^. Leivonen *et al*. reported the first nationwide pseudo-prospective ( *i.e.*, based on a Finnish national health registry) study of prenatal maternal smoking and TS ^37^. In this sample, the hypothesized association of maternal smoking with TS *per se* was not significant, but maternal smoking during pregnancy was significantly associated with combined TS and ADHD, and the odds ratio for TS+ADHD following first trimester smoking was 4.0 (95% CI 1.2–13.5, p=.027). This association provides only modest support for the link between prenatal toxicity and TS. However, this study is consistent with the idea shared by many tic experts that the current nosology artificially separates tics from other common symptoms in people with TS. Similarly, in a large case-control report from the Tourette International Collaborative Genetics study, perinatal complications were more common in the TS patients, as were OCD and ADHD ^38^.

### Pathophysiology

***Animal models.*** A face-valid animal model of tics has been a very important but elusive goal ^39^. McCairn and colleagues have now published their important work characterizing a nonhuman primate tic model ^40^. Temporary unilateral disinhibition of the nucleus accumbens (NA)—the ventral, more limbic-connected part of the striatum—produces vocalizations in monkeys that resemble vocal tics in human patients. The vocalizations sometimes were associated with local field potential (LFP) spikes in the NA, but not always; in the absence of local LFP spikes, phase coupling in the alpha frequency band between NA, anterior cingulate and primary motor cortex tended to be stronger. By contrast, disinhibition of the (more traditionally motor) dorsolateral putamen led to repetitive movements that the authors call “myoclonic tics,” whose phenomenology is less similar on its face to that of tics, and which were regularly preceded by LFP spikes.

Vasoactive intestinal peptide-expressing interneurons were targeted for developmental injury using a conditional expression model in mice ^41^. These mice showed hyperactivity and altered firing of some cells at the onset of the transition from quiescence to locomotion. How this may relate to lower numbers of parvalbumin-containing interneurons in TS ^42^ is unclear. An interesting contrast based on the parvalbumin data is provided by a model in which fast-spiking interneurons in the dorsal striatum were ablated ^43^. These mice developed anxiety and increased frequency of grooming rituals when exposed to an acute stressor, namely, repeated unpredictable bursts of acoustic white noise. They did not show increased stereotypies in response to amphetamine.

In another rodent model, D1CT-7 mice—in which dopamine D1 receptors are targeted— were compared to wild-type mice ^44^. Spatial confinement triggered stress reflected by corticosterone release in both groups, but exacerbated abnormal behaviors in the D1CT-7 mice, such as digging, biting, jumping and motor perseveration. Stress also led to impaired prepulse inhibition, thought of as reflecting sensorimotor gating. Both the abnormal motor behavior and the prepulse inhibition deficit improved with clonidine, haloperidol, or SCH23390 (a dopamine D1 receptor antagonist). Collectively, these results support the potential utility of this model in screening new treatments for tic disorders.

Finally, spontaneously-occurring models of tics in other animals are of great interest ^45^. Kalueff and colleagues provide a helpful review of the substantial body of knowledge available about stereotyped grooming patterns in rodents ^46^, arguing appropriately that these patterns may be “useful for understanding the neural circuits that are involved in complex sequential patterns of action” in human disease, including in TS ^47^.

***Electrophysiology.*** In contrast to findings from previous studies in older patients, resting motor threshold and variability of motor evoked potential responses in 17 TS subjects, age 12–22, were increased versus controls, while the gain of motor excitability was reduced ^48^. The authors suggest that these findings may relate to delayed maturation of the cortical-cortical and corticospinal motor networks in TS.

Local field potentials (LFP) in the posterolateral globus pallidus pars interna (GPi) were recorded at rest and during tics and normal voluntary movements in three adults with TS during deep brain stimulation surgery and compared to GPi LFP recordings in four Parkinson disease patients off medication ^49^. Tics were associated with increased gamma (35–200 Hz) and high frequency (200–400 Hz) activity, and with cross-frequency coupling (between the phase of beta band activity and the amplitude of the high-frequency oscillations).

Negative reinforcement learning is a key variable in theories of behavior therapies proven effective for TS. In this context, the findings of a 2016 report from Nottingham, England, are interesting. Behavioral and event-related potential data were collected during the learning of stimulus-response pairs using positive or negative reinforcement ^50^. Participants included children age 9–17 with TS, ADHD, both or neither. Lower accuracy and smaller P3 amplitudes were observed in association with ADHD, but not TS.

O’Connor and colleagues have developed a cognitive-behavioral therapy approach to tic treatment, discussed below in the *Treatment* section, based in part on electrophysiological findings. For instance, previous studies had identified a reduced P300 oddball effect in TS patients with or without OCD. This group has now reported a study of event-related potentials in adults with TS or a body-focused repetitive behavior disorder (such as trichotillomania) in comparison to a healthy control group ^51^. The P300 oddball effect was lower in both patient groups before treatment, and increased with cognitive-psychophysiological treatment.

***Neuroimaging studies.*** A structural brain MRI study in 103 children with TS, age 7–17 years, and 103 matched control subjects, found greater gray matter volume in TS in the posterior thalamus, hypothalamus and midbrain, and decreased white matter volume in orbital prefrontal and anterior cingulate cortex ^52^. Structural abnormalities in these regions were hypothesized to influence somatosensory processing, decision making or reinforcement learning. A diffusion tensor imaging study of 27 children with TS and 27 matched controls found decreased fractional anisotropy and increased radial diffusivity in a number of white matter tracts in the TS group, and some of these findings correlated with symptom severity ^53^. 

Eddy and colleagues continued their research using theory of mind (ToM) approaches to TS, reporting on an fMRI study involving a task that required the subject to infer another person’s thoughts when making decisions ^54^. TS subjects showed overall lower responses to the ToM task and to a control task, but also in several regions a lower increase with the ToM task compared to the control task. These regions included the posterior cingulate, right angular gyrus and right amygdala, regions involved in previous ToM studies in people without tics. Group differences correlated with various symptom severity measures, including a strong correlation of task-related temporoparietal junction activation with premonitory urge severity.

SPECT imaging with a perfusion marker was used to investigate the possible mechanism of action of deep brain stimulation in the internal GPi and in the medial thalamus ^55^. Controls included sham stimulation in the TS group and unstimulated subjects without tics. Active stimulation in either site decreased regional blood flow in basal ganglia and cerebellum and increased perfusion to the frontal cortex, including the supplementary motor area.

Brain imaging studies include very large data sets, and have required development of novel statistical methods. Lessov-Schlaggar *et al.* argued thoughtfully that typical statistical analyses of a single variable at a time may be inadequate to find many important solutions to “complex disorders of brain development and function”, such as TS ^56^.

***Pharmacological studies.*** German researchers reported a fascinating study in 37 medication-free adults with TS and 36 controls using magnetic resonance spectroscopy (MRS) ^57^. The study tested the hypothesis that GABA, glutamine (Gln) and glutamate (Glu) concentrations in striatum and thalamus would differ in people with TS. Glutamine, Gln+Glu and the Gln:Glu ratio were lower in TS, more so in the striatum than in the thalamus. The striatal Gln concentration correlated negatively with tic severity on the day of the scan, and thalamic Glu concentration correlated negatively with premonitory urge severity. Surprisingly, these abnormalities tended to improve during treatment with aripiprazole. The authors work hard to minimize any effects of head motion, which might otherwise confound these results. The results overall suggest an abnormality in glutamatergic transmission in TS. Readers who are not MRS gurus may benefit from examining their Supplementary Figure 5, which demonstrates the challenges in eking out signal for these compounds.

Gremel *et al.* ^58^ present data from an optogenetic study in mice, showing that removing cannabinoid type 1 (CB1) receptors from orbitofrontal cortex efferents to dorsal striatum prevents the mice from graduating from intentional to habitual lever pressing. The authors conclude that this cannabinoid-modulated orbitofrontal-striatal pathway is essential to the development of habits. As tics may be conceptualized as involving overenthusiastic habit formation circuitry, one might have expected the opposite result, given apparent beneficial short-term effects of cannabinoids on tic severity ^59^.

In another mouse model, knocking out the histamine H3 receptor impaired prepulse inhibition, and abolished or reduced dopaminergic signaling via both D1 and D2 receptors ^60^. These results, combined with previous studies linking histamine to TS, support the development of histamine H3 receptor ligands for possible use in TS.

***Clinical and neuropsychological studies.*** One interesting study focused on the clue that classic features of TS include echopraxia and suggestibility (the triggering of a tic by discussing or seeing it). Following up on their previous work ^61, 62^, Münchau and colleagues asked tic-free human control participants to imitate separately a tic and another, non-tic facial movement from a matched participant with TS ^63^. While doing so, subjects watched video of one of the two movements. For non-tic movements, TS subjects responded like controls, in that movements were performed more quickly when the video was congruent with the requested movement than when it was incongruent. However, when asked to execute a tic, TS subjects responded equally quickly regardless of the video content. The authors conclude that “tic-like movements do not occur as a consequence of a failure to inhibit motor output. Instead, tics might be considered highly overlearned behavior that can be triggered without interference by external, incompatible movement stimuli.”

Most adults with TS feel that at least some of their tics are an eventual voluntary capitulation to an almost irresistible urge, though some tics are felt to be more truly involuntary. Researchers from Paris reported an interesting study of how adults with TS judge agency (the sense that one initiates and causes one’s own actions) ^64^. The study involved manipulating in several different ways whether a participant’s hand movements were accurately reflected in the movements of a computer cursor. Those with TS recognized when their intended movements were substantially altered, yet were less aware that their intentions had been altered when the alterations comprised enhancement of their performance. These results add to a prior study of agency in adolescents with TS, in which awareness of their intention to move was earlier in those with greater tic suppression ability and later in those with more severe premonitory urges ^65^. Together, these studies suggest that the sense of agency with intentional movement differs subtly in people with TS, and may relate to patients’ judgments of whether their tics are volitional.

### Treatment

A meta-analysis of tic treatment discusses patient satisfaction in addition to efficacy and side effects, and concludes that there is good evidence supporting a favorable benefits:harms ratio for the most commonly used medications, but that “larger and better-conducted trials addressing important clinical uncertainties are [still] required” ^66^.

***Psychological interventions.*** Behavior therapy is now an accepted first-line treatment for tic disorders. Still, some practitioners retain misconceptions about its safety. A report on 228 participants in randomized controlled trials (RCTs) of Comprehensive Behavioral Intervention for Tics (CBIT) helps to confute those concerns ^67^. Specifically, CBIT participants were no more likely to have new tics, have adverse events, increase tic medications or have an exacerbation in psychiatric symptoms relative to patients who received supportive therapy. In these studies, 45% of the participants randomized to CBIT were treatment responders, versus only 13% of those assigned to the control therapy.

Factors that slow wider implementation of behavior therapies for tics include cost and a limited number of trained practitioners. Group (rather than individual) therapy is one option that could address these obstacles. A small study provided initial testing of this possibility using a treatment and a control group. Both groups had improvements in quality of life, and the greatest improvements in motor tic severity occurred in the HRT group ^68^. The most important conclusion from this pilot study was that the group format was acceptable and appears not to impair efficacy.

Another potential solution to the implementation concerns noted above is providing therapy online. Müller-Vahl and colleagues are beginning a large RCT that will test the efficacy of CBIT delivered by internet ^69^. Ricketts and colleagues completed an RCT of CBIT delivered by VoIP (voice over internet protocol) compared to a wait list; the active treatment condition was significantly more successful in reducing tics, with one third of patients judged to be responders ^70^. Another approach is to implement CBIT outside the mental health setting, demonstrated by an initial feasibility study from neurology and developmental pediatrics offices ^71^.

Research continues on other psychotherapy approaches. Suppressing tics is generally uncomfortable—leading to the theory of negative reinforcement that motivates CBIT and exposure and response prevention approaches to tic treatment—and in recent years Acceptance and Commitment Therapy for various conditions has become more popular, and pilot studies have appeared applying this approach to tics ^72, 73^. In this setting, Gev *et al.* ^74^ report their study of 45 children and adolescents with TS who rated severity of tic urges, and were observed, during each of three 2-minute conditions: baseline (ticcing freely), tic suppression, and urge acceptance. Urges and discomfort decreased significantly during the acceptance condition, and more importantly, so did tic frequency. By contrast, tic urge intensity and self-rated discomfort increased during the tic suppression condition. Interpreting the results is complicated by the fact that the acceptance condition followed a brief relaxation intervention. These findings suggest that acceptance therapy may be better—accepted—by patients. It is now ripe for tests of long-term efficacy.

For some years, O’Connor, Leclerc and colleagues have developed and utilized a cognitive-behavioral-psychophysical treatment model for tic disorders. The treatment aims to address sensorimotor function more broadly, including proprioception, response inhibition and perfectionism, rather than focusing on tics directly. In 2016, several important publications from their group addressed this approach, including an open study of 102 adults with a chronic tic disorder ^75^, in which tic severity improved for both simple and complex tics, and the improvement remained at 6 months. Measures of self-esteem and perfectionism also improved. A pilot study in children age 8–16 was also reported ^76^, followed by initial results with a manualized version of this therapy in children age 8–12 ^77^. This “cognitive-psychophysiological treatment” model provides another potential approach to treatment that may prove to have more generalizable benefits.

Specht and colleagues studied a home-based, parent-administered behavior therapy for primary complex motor stereotypies ^78^. Parents reported decreases of 15% to 24% in stereotypy severity at 1, 2 and 3 months after they received the instructional DVD. This open study is important not only for patients with (non-tic) stereotypies, but also for its innovative treatment delivery and study design.

***Medication.*** An open-label, rater-blind, 8-week study of deutetrabenazine in TS provided positive results ^79^. This compound is a deuterium-substituted tetrabenazine (a VMAT2 inhibitor) with slower elimination half-life, and was approved by the U.S. Food and Drug Administration (FDA) in 2017 as a treatment for chorea in Huntington disease, making it potentially available for off-label use for tics. Similarly, another VMAT 2 inhibitor, valbenazine, was approved in 2017 for treatment of tardive dyskinesia.

Adding to prior evidence of anti-tic efficacy, a prospective, open case series in 44 adults with TS found that aripiprazole helped non-tic symptoms as well as tics, though premonitory urges to tic were not significantly improved ^80^. However, in May 2016, the FDA warned that aripiprazole can cause disinhibition in the form of impulse control disorders ^81^. Similar side effects have been described for levodopa and dopamine agonists, but had not been recognized for aripiprazole, which is a partial agonist at dopamine receptors.

Other medication trials reported in 2016 include a failed add-on RCT with N-acetyl-cysteine ^82^ and a case series suggesting iron supplementation may improve tics in TS patients with low serum ferritin ^83^.

***Neurosurgery.*** Surgery is an option for carefully selected patients with TS, yet several important questions remain ^84^. The Galeazzi Institute in Milan, Italy, provided a progress report on their large sample of TS patients treated with deep brain stimulation (DBS), mostly in the ventromedial thalamus ^85^. In 11 of 48 patients (23%), the device was removed after “inflammatory complications” or poor compliance with follow-up. In the remaining 37 patients, 29 had a more than 50% reduction in YGTSS scores (clinician-rated tic severity and impairment). In a separate publication, they argued that the patient’s symptoms beyond tics should be considered when a DBS target is selected ^86^. Hartmann argued contrariwise that our current state of knowledge better supports a narrower focus on tic reduction in choosing a DBS target ^87^.

The TS group from Maastricht, The Netherlands, reported positive unblinded follow-up results in five patients with refractory TS treated with DBS in the anterior GPi ^88^, and a Chinese group reported unblinded 1-year follow-up of GPi DBS in 24 patients with TS, with improvement, on average, in both tics and OCD symptoms (50% reduction in mean YGTSS total tic score and 36% reduction in mean Y-BOCS score; Supplementary File 1) ^89^. Interestingly, this latter report includes one patient whose tic improvement continued after the DBS electrode was removed (p. 1025), consistent either with spontaneous improvement over time or with a micropallidotomy lesion effect.

DBS in a mouse model suggests that self-injurious behaviors, probably over-represented in TS patients referred for surgery, may be amenable to DBS of the subthalamic nucleus ^90^.

Given the current lack of consensus on DBS methods in TS, gathering data on all DBS patient outcomes in TS is crucial, and a recent collaborative report described the establishment of the International Deep Brain Stimulation Registry and Database for Gilles de la Tourette syndrome ^91^.

***Other treatment.*** Many patients have reported to their doctor that music performance reduces their tic symptoms. Brown ^92^ addressed this formally, surveying 183 musicians diagnosed with TS. On a scale of 1 (drastic symptom worsening) to 5 (drastic symptom improvement), subjects reported a mean of 4.45 for the effect of engaging in a musical activity (performance, not passive listening). This result strongly supports the patient anecdotes and suggests that formal music therapy could be tested in a randomized controlled trial for benefit on TS symptoms.

Some tic patients have been very interested in PANDAS, a name representing the hypothesis that obsessions, compulsions and tics represent an aberrant immune response to streptococcal infection in some children ^93^. Recently a second double-blind study of intravenous immunoglobulin (IVIG) for OCD symptoms in PANDAS patients was published ^94^. Unfortunately, although effects during the open label phase were substantial, there was little difference between the IVIG and control groups during the double-blind portion of the study.

### Tics, family and society

Three tic experts contributed a thoughtful review of the effect of tics and non-tic symptoms (or comorbidities, depending upon one’s nosological viewpoint) on social relationships and quality of life in TS ^95^. Wadman and colleagues performed an in-depth study of 35 youth with TS, their parents, and school personnel to understand what problems TS caused at school ^96^. Their conclusion will seem sadly familiar to TS patients and clinicians: “Young people and parents agreed more strongly with each other than they did with staff regarding school difficulties faced by individuals, and staff generally reported fewer TS-related difficulties.”

### Additional reference sources

The first Frontiers Research Topic on TS was published this year, including over 30 articles on TS, many stemming from the 2015 conference in London ^97^. Thenganatt and Jankovic provided a useful review of TS ^98^. A comprehensive review of Provisional Tic Disorder also appeared ^99^; this diagnosis refers to children whose first tic was less than a year ago, similar to Transient Tic Disorder in earlier nosologies. A helpful overview of TS genetics ^100^, and a review of epigenetic mechanisms and how they may prove to affect TS, both appeared in mid-2016 ^101^.

## Conclusions

Advances in many areas of TS research and clinical care were reported in 2016. Smaller pilot studies remain important for purposes other than settling a question: they can be useful for testing feasibility, for improving methods, and for identifying new hypotheses. (In fact, one of the most significant publications in TS history was a single-blind *N*-of-1 study ^102^.) Several of the studies mentioned above are small studies that fulfill one or more of these goals.

Nevertheless, as the title of the 2016 Frontiers Research Topic suggests, TS research is beginning to grow from case series and small pilot studies into finding “new avenues through large-scale collaborative projects.” This change is important, especially in light of increasing recognition that reproducibility is an important concern in all of science, and clearly in neuroscience ^103, 104^. Larger samples are important to addressing this concern, and give greater hope for important new discoveries in the future.

## Supplemental material

Supplementary File 1: Calculation of the percent improvement numbers from the Zhang *et al.* DBS report ^89^.
