# Supplementary material for: Tourette syndrome research highlights from 2016
Source: F1000Res. 2017 Nov 29;6:1430. Originally published 2017 Aug 11. [Version 2] doi: 10.12688/f1000research.12330.2 (PMC5621096; doi:10.12688/f1000research.12330.2)
Supplement: Peer review history

# Peer review: Referee response for version 2

**DOI:** 10.5256/f1000research.14399.r28529

**Published:** 2017-11-30

**Authors:** James F. Leckman

**Affiliations:** 1 Child Psychiatry, Psychiatry, Psychology and Pediatrics, Yale Child Study Center, Yale School of Medicine, New Haven, CT, USA

Thank you for the clarification.

I have read this submission. I believe that I have an appropriate level of expertise to confirm that it is of an acceptable scientific standard.

# Peer review: Referee response for version 1

**DOI:** 10.5256/f1000research.13348.r25613

**Published:** 2017-10-04

**Authors:** Christos Ganos

**Affiliations:** 1 Department of Neurology, Charité, University Medicine Berlin, Berlin, Germany

I thoroughly enjoyed the "Tourette Syndrome research highlights from 2016" article by Prof. Black.

 It is a very well organised effort to present a succinct summary of relevant research from that year and I would like to congratulate Prof. Black for his dedication in putting this together. 

 I only have some minor suggestions.

1. It might be thematically more suitable to integrate the first paragraph of the sensory findings of the "Clinical and neuropsychological studies" ["Thresholds for externally applied sensory... ...or central sensorimotor processing"] to the "Premonitory Urges" section, as indeed these refer to the same topic.
2. On the reference of the mouse model of Xu et al. ["These mice developed anxiety and increased frequency of grooming rituals."] it might be of relevance to highlight that the abnormal behaviours only occurred after exposure to environmental stressors. 
3. On the Treatments/psychological interventions section, it could be relevant to mention that CBIT responders in those trials as measured by the CGI-I were 45% of the total sample. [Data from the cited publication: "CBIT were significantly more likely to be classified as treatment responders on the CGI-I (child = 53%, adult = 38%, combined sample = 45%)"]

I have read this submission. I believe that I have an appropriate level of expertise to confirm that it is of an acceptable scientific standard.

# Response

**Published:** 2017-10-04

**Authors:** Kevin J Black

**Affiliations:** Department of Psychiatry, Washington University in St Louis, USA

Thanks for the kind comments and expert suggestions, which I can include in a revision.

# Peer review: Referee response for version 1

**DOI:** 10.5256/f1000research.13348.r25611

**Published:** 2017-09-28

**Authors:** Davide Martino

**Affiliations:** 1 University of Calgary, Calgary, AB, Canada

The authors present an interesting and comprehensive review of highlighted articles published in 2016 on Tourette syndrome. The article is very well written.

 There are a few articles that might also be considered for highlight, in addition to those already referenced.

 Comorbidities:

- Johnco C et al. (2016) ^1^
  This is particularly relevant in view also of an important finding coming from a 2017 population-based study (Fernandez de la Cruz, Biol Psychiatry 2017)

Genetics:

- Bertelsen B et al. (2016) ^2^

Environmental factors (etiology):

- Abdulkadir M et al. (2016) ^3^

Imaging:

- Wen H et al. (2016) ^4^
- Liao W et al. (2017) ^5^

I have read this submission. I believe that I have an appropriate level of expertise to confirm that it is of an acceptable scientific standard.

## References

[1] JohncoCMcGuireJFMcBrideNMMurphyTKLewinABStorchEA: Suicidal ideation in youth with tic disorders. *J Affect Disord*.2016;200: 10.1016/j.jad.2016.04.027 204-11 10.1016/j.jad.2016.04.027 27136419PMC4887311

[2] BertelsenBStefánssonHRiff JensenLMelchiorLMol DebesNGrothCSkovLWergeTKaragiannidisITarnokZBartaCNagyPFarkasLBrøndum-NielsenKRizzoRGulisanoMRujescuDKiemeneyLATosatoSNawazMSIngasonAUnnsteinsdottirUSteinbergSLudvigssonPStefanssonKKussAWPaschouPCathDHoekstraPJMüller-VahlKStuhrmannMSilahtarogluAPfundtRTümerZ: Association of AADAC Deletion and Gilles de la Tourette Syndrome in a Large European Cohort. *Biol Psychiatry*.2016;79(5) : 10.1016/j.biopsych.2015.08.027 383-91 10.1016/j.biopsych.2015.08.027 26444075

[3] AbdulkadirMTischfieldJAKingRAFernandezTVBrownLWCheonKACoffeyBJde BruijnSFElzermanLGarcia-DelgarBGilbertDLGriceDEHagstrømJHedderlyTHeymanIHongHJHuyserCIbanez-GomezLKimYKKimYSKohYJKookSKupermanSLamerzALeventhalBLudolphAGMadruga-GarridoMMarasAMesschendorpMDMirPMorerAMünchauAMurphyTLOpenneerTJPlessenKJRathJJRoessnerVFründtOShinEYSivalDASongDHSongJStolteAMTübingJvan den BanEVisscherFWandererSWoodsMZinnerSHStateMWHeimanGAHoekstraPJDietrichA: Pre- and perinatal complications in relation to Tourette syndrome and co-occurring obsessive-compulsive disorder and attention-deficit/hyperactivity disorder. *J Psychiatr Res*.2016;82: 10.1016/j.jpsychires.2016.07.017 126-35 10.1016/j.jpsychires.2016.07.017 27494079PMC5026935

[4] WenHLiuYWangJRekikIZhangJZhangYTianHPengYHeH: Combining tract- and atlas-based analysis reveals microstructural abnormalities in early Tourette syndrome children. *Hum Brain Mapp*.2016;37(5) : 10.1002/hbm.23146 1903-19 10.1002/hbm.23146 26929221PMC6867590

[5] LiaoWYuYMiaoHHFengYXJiGJFengJH: Inter-hemispheric Intrinsic Connectivity as a Neuromarker for the Diagnosis of Boys with Tourette Syndrome. *Mol Neurobiol*.2017;54(4) : 10.1007/s12035-016-9863-9 2781-2789 10.1007/s12035-016-9863-9 27011382

# Response

**Published:** 2017-09-28

**Authors:** Kevin J Black

**Affiliations:** Department of Psychiatry, Washington University in St Louis, USA

Thanks for your input. These are excellent suggestions and I plan to include them in a revision.

# Response

**Published:** 2017-11-22

**Authors:** Kevin J Black

**Affiliations:** Department of Psychiatry, Washington University in St Louis, USA

We cited the Bertelsen et al paper in our 2015 highlights paper [1]. The Liao et al paper [2] uses a relatively lenient threshold for censoring head motion [3], complicating interpretation. I've included your other suggestions in version 2.

[1] Richards CA and Black KJ. Tourette syndrome research highlights 2015 [version 1; referees: 3 approved]. *F1000Research* 2016, **5**:1493 

(doi: 10.12688/f1000research.8769.1).

[2] Liao W, Yu Y, Miao HH, Feng YX, Ji GJ, Feng JH: Inter-hemispheric Intrinsic Connectivity as a Neuromarker for the Diagnosis of Boys with Tourette Syndrome. *Mol Neurobiol*. 2017; **54** (4): 2781-2789 PubMed Abstract | Publisher Full Text

[3] Power JD, Mitra A, Laumann TO, Snyder AZ, Schlaggar BL, Petersen SE: Methods to detect, characterize, and remove motion artifact in resting state fMRI. *Neuroimage* 2014; **84**:320-341 (doi: 10.1016/j.neuroimage.2013.08.048)

# Peer review: Referee response for version 1

**DOI:** 10.5256/f1000research.13348.r25610

**Published:** 2017-09-12

**Authors:** James F. Leckman

**Affiliations:** 1 Child Psychiatry, Psychiatry, Psychology and Pediatrics, Yale Child Study Center, Yale School of Medicine, New Haven, CT, USA

Prof. Kevin Black has provided an up-to-date and largely comprehensive summary of the current status of our knowledge concerning the phenomenology, natural history, etiology, pathophysiology, and treatment of Tourette syndrome (TS). However, one important area that is largely missing concerns the findings from post-mortem studies of the brains of individuals with TS that have been conducted by Flora Vaccarino and her laboratory at Yale University. The most notable and highly-cited article from 2016 presents an analysis of the transcriptome of striatal tissue (caudate and putamen) from the brains of nine TS and nine matched normal control subjects (Lennington et al., 2016 ^1^). The authors found 309 down-regulated and 822 up-regulated genes using a data-driven gene network analysis. More specifically, they identified 17 gene co-expression modules associated with TS. The top-scoring down-regulated module in TS was enriched for striatal interneuron transcripts. This finding confirmed earlier studies by the Vaccarino laboratory that had reported decreased numbers of cholinergic and gamma-aminobutyric acidergic interneurons in the same brain regions (Kalathini et al., 2005; Kataoka et al., 2010 ^2^). However, the top-scoring up-regulated module was enriched in immune-related genes, consistent with activation of microglia in patients’ striatum. While these findings confirm the earlier post-mortem studies using unbiased stereological techniques, they also point to the important role of neuroinflammation in TS pathophysiology. Indeed, a deeper understanding of neuroimmunology may well transform the field of neuropsychiatry and point to novel treatment approaches to TS and related disorders (Leckman & Vaccarino, 2015 ^3^).

I have read this submission. I believe that I have an appropriate level of expertise to confirm that it is of an acceptable scientific standard, however I have significant reservations, as outlined above.

## References

[1] LenningtonJBCoppolaGKataoka-SasakiYFernandezTVPalejevDLiYHuttnerAPletikosMSestanNLeckmanJFVaccarinoFM: Transcriptome Analysis of the Human Striatum in Tourette Syndrome. *Biol Psychiatry*.2016;79(5) : 10.1016/j.biopsych.2014.07.018 372-82 10.1016/j.biopsych.2014.07.018 25199956PMC4305353

[2] KataokaYKalanithiPSGrantzHSchwartzMLSaperCLeckmanJFVaccarinoFM: Decreased number of parvalbumin and cholinergic interneurons in the striatum of individuals with Tourette syndrome. *J Comp Neurol*.2010;518(3) : 10.1002/cne.22206 277-91 10.1002/cne.22206 19941350PMC2846837

[3] LeckmanJFVaccarinoFM: Editorial commentary:. *Brain Res*.2015;1617: 10.1016/j.brainres.2014.09.052 1-6 10.1016/j.brainres.2014.09.052 25283746

# Response

**Published:** 2017-09-12

**Authors:** Kevin J Black

**Affiliations:** Department of Psychiatry, Washington University in St Louis, USA

I agree with the importance of the Lennington et al., 2016, paper. ^1^ It is absent from the "from 2016" highlights article only because (since the final version appeared online before its official publication date) we discussed it in the 2015 highlights article ^2^ (first paragraph under Pathophysiology). We cited the Kataoka et al publication ^3^ in the 2014 highlights paper. ^4^ I do appreciate the reference to the 2015 editorial, ^5^ which together with your review above better highlights the relevance of the Lennington et al. report to the broader question of neuroimmunology in Tourette syndrome.

1. Lennington JB, Coppola G, Kataoka-Sasaki Y, Fernandez TV, Palejev D, Li Y, Huttner A, Pletikos M, Sestan N, Leckman JF, Vaccarino FM: Transcriptome Analysis of the Human Striatum in Tourette Syndrome. *Biol Psychiatry*. 2016; **79** (5): 372-82 PubMed Abstract | Publisher Full Text 
2. Richards CA and Black KJ. Tourette syndrome research highlights 2015 [version 1; referees: 3 approved]. *F1000Research* 2016, **5**:1493 (doi: 10.12688/f1000research.8769.1)
3. Kataoka Y, Kalanithi PS, Grantz H, Schwartz ML, Saper C, Leckman JF, Vaccarino FM: Decreased number of parvalbumin and cholinergic interneurons in the striatum of individuals with Tourette syndrome. *J Comp Neurol*. 2010; **518** (3): 277-91 PubMed Abstract | Publisher Full Text
4. Richards CA and Black KJ. Tourette Syndrome research highlights 2014 [version 2; referees: 1 approved, 2 approved with reservations]. *F1000Research* 2015, **4**:69 (doi: 10.12688/f1000research.6209.2)
5. Leckman JF, Vaccarino FM: Editorial commentary:. *Brain Res*. 2015; **1617**: 1-6 PubMed Abstract | Publisher Full Text

# Peer review: Referee response for version 1

**DOI:** 10.5256/f1000research.13348.r24972

**Published:** 2017-08-29

**Authors:** Keith A. Coffman

**Affiliations:** 1 Division of Pediatric Neurology, Department of Pediatrics, Children's Mercy Hospital, Kansas City, MO, USA

The manuscript submitted by Dr. Black is a very thorough and comprehensive review of the recent literature on Tourette Syndrome. It is exceptionally well written and organized. 

 There is one error that needs to be corrected. In the following sentence, there is a missing word and simply a citation number.

*"Another potentially useful approach to TS genetic research was illustrated by ****28, who sequenced candidate genes suggested by previous research in order to identify potential functional variants linked to TS."*

 Other than this, I could find no errors or modifications that I would recommend before publishing this manuscript.

I have read this submission. I believe that I have an appropriate level of expertise to confirm that it is of an acceptable scientific standard.

# Response

**Published:** 2017-08-29

**Authors:** Kevin J Black

**Affiliations:** Department of Psychiatry, Washington University in St Louis, USA

Thank you! I can correct the missing author name in a revision after other reviews are back.
